# Plant-Derived Colorants for Food, Cosmetic and Textile Industries: A Review

**DOI:** 10.3390/ma14133484

**Published:** 2021-06-23

**Authors:** Patrycja Brudzyńska, Alina Sionkowska, Michel Grisel

**Affiliations:** 1 Department of Biomaterials and Cosmetics Chemistry, Faculty of Chemistry, Nicolaus Copernicus University in Torun, Gagarin 7 Street, 87-100 Torun, Poland; alinas@umk.pl; 2 Chemistry Department, UNILEHAVRE, FR 3038 CNRS, URCOM EA3221, Normandie University, 76600 Le Havre, France; michel.grisel@univ-lehavre.fr

**Keywords:** plant-derived colorants, anthocyanins, isoprenoids, betalains, cosmetic, textile, food coloration

## Abstract

This review provides a report on properties and recent research advances in the application of plant-derived colorants in food, cosmetics and textile materials. The following colorants are reviewed: Polyphenols (anthocyanins, flavonol-quercetin and curcumin), isoprenoids (iridoids, carotenoids and quinones), N-heterocyclic compounds (betalains and indigoids), melanins and tetrapyrroles with potential application in industry. Future aspects regarding applications of plant-derived colorants in the coloration of various materials are also discussed.

## 1. Introduction

**Figure 1:**
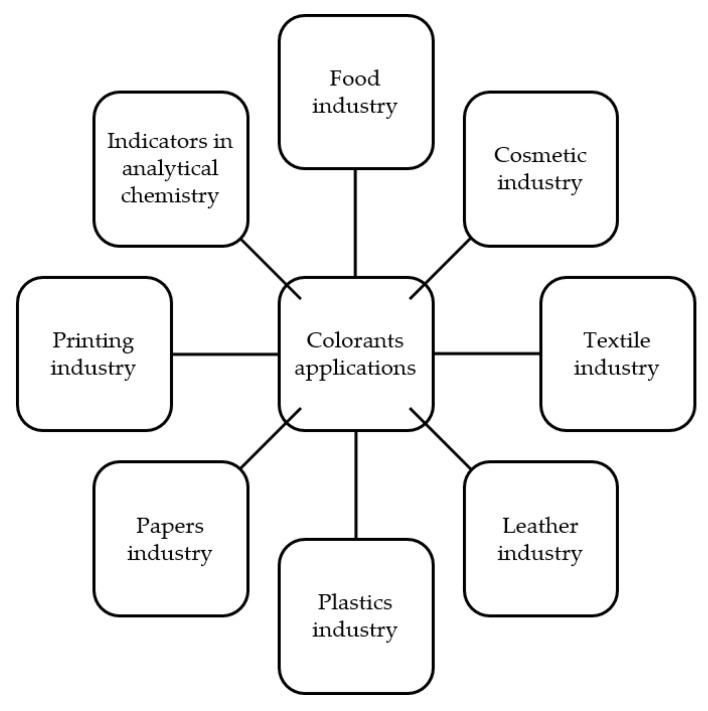
The exemplary industrial application of colorants.

There is currently a revival in the application of natural ingredients that can be observed in different areas of human lives. This revival concerns not only phytotherapy, but also the need to create various products based on natural raw materials, including plant-derived ingredients. All industries are becoming more ecological, less harmful to the environment and healthier for consumers. One example of the extensive utilization of natural raw materials currently observed is the broad use of many herbs, vegetable oils or essential oils in different products. There is also a group of very important compounds—the colorants that are indispensable in various applications. Colorants provide the desired color and thus make it possible to obtain satisfying products for consumers in a wide variety of domains, such as the textile, food or cosmetic industries. Nowadays, most of the production of all colorants is consumed by the textile industry, but they are also used in other industries like paper, leather, printing, plastics and as indicators in chemistry, as shown in Figure 1. 

The entire market for colorants is highly specialized and can be perceived as closed, but what drives the market is the consumer’s desire to use natural rather than synthetic colorants. Therefore, the global market of natural colorants is growing faster than the entire color market. This trend is caused by their use in the food industry [1]. Plant-derived colorants are successfully used in food; on a larger scale they can have the potential to be more frequently used in cosmetics or textiles. Nowadays, in the textile industry plant-derived colorants are rather used only in craft dyeing. In cosmetic applications, from the list of 153 compounds included on the EU-positive list of dyes and pigments [2], approximately 8% are plant extracts, 20% are inorganic pigments and 72% are synthetic dyes and pigments—thus the majority is still made up by synthetic or mineral ones. Nowadays, plant-derived dyes that can be used in cosmetics, according to applicable regulations, include, among others, β-carotene, canthaxanthin, annatto, lycopene, curcumin, vegetable carbon, riboflavin, caramel, pepper extract, capsanthin, capsorubin, beetroot red and a variety of anthocyanins (cyanidin, peonidin, malvidin, delphinidin, petunidin, pelargonidin).

Besides the fact that plant-derived colorants are biodegradable and compatible with the environment, they are also less allergenic and less toxic than synthetic ones. Due to harmful effects, some of the latter were banned in different countries. Apart from their dying ability, plant-derived colorants can also be relevant for their additional properties, such as their antioxidant and antimicrobial activity, among others, which can be another strong benefit for their application in the cosmetic, textile and food industry [3]. Plan-derived colorants belong to different chemical groups like carotenoids, polyphenols, quinones or alkaloids. Due to their chemical structure, they are unstable under the influence of high temperature, light and different pH values. This leads to research and innovation intending to improve the stability of colorants obtained from plants. To provide a solution to increase colorants, stability technologies such as nanotechnology and encapsulation bring significant improvements [1]. Nevertheless, the instability of plant-derived colorants when applied in products is not the only problem. It is important to be aware of different parameters that have an impact on color, for instance the interaction of components: In the case of cosmetics and food, the impact of packaging and shelf-life requirements. Other issues concern their presentation in a product—as a liquid, powder, paste or dispersion [4]. Additionally, color from plant sources may also depend on climate conditions, which may lead to difficulties in maintaining consistent quality [5]. Finally, botanical colorants are more expensive than synthetic ones, with a cost that can be two to ten times higher compared to synthetic colorants [6]. To lower the production costs of natural colorants, agro-food byproducts could be applied, which contributes to a reduction in wastes. Even though this would be an ecological approach, modern usage of byproducts is limited by regulations [7]. It is necessary to improve the price-to-performance ratio, this being indispensable to increase their industry application [1]. It is generally easier to obtain diverse colors with synthetic colorants than with plant-derived ones unless different groups of colorants are mixed. Another point is that manufacturing synthetic colorants requires a large number of different compounds, as well as several stages and reactions. Furthermore, the history of the application of plant-derived dyes prevails in their favor. Historically, those colorants were almost the only coloring agents that society used for the textile, leather, paper, inks and art fields, among others. Valuable dye plants included turmeric, indigo, madder, woad or logwood. Nevertheless, with both chemical development and the discovery of synthetics, the plant-derived colorants were forgotten. The first synthetic colorant was mauve, discovered by William Henry Perkin in 1856 [4]. This marked the beginning of changes in the dyeing industry, and natural colorants obtained from plants and animals were progressively substituted by synthetic ones. Subsequent discoveries and experiments led to the manufacturing of organic pigments and the era of synthetic colorants began. Scientists focused not only on creating artificial colorants but also on the re-synthesis of natural dyes. The first to be obtained was alizarine, and then indigo in 1880 [4]. Alizarin was synthesized by Graebe and Lieberman in the year 1868, and this method became useful in industry and completely replaced products obtained from the plant *Rubia tinctorum*. The consequences resulting from such discoveries were widespread and changed standard practices [8]. 

**Figure 2:**
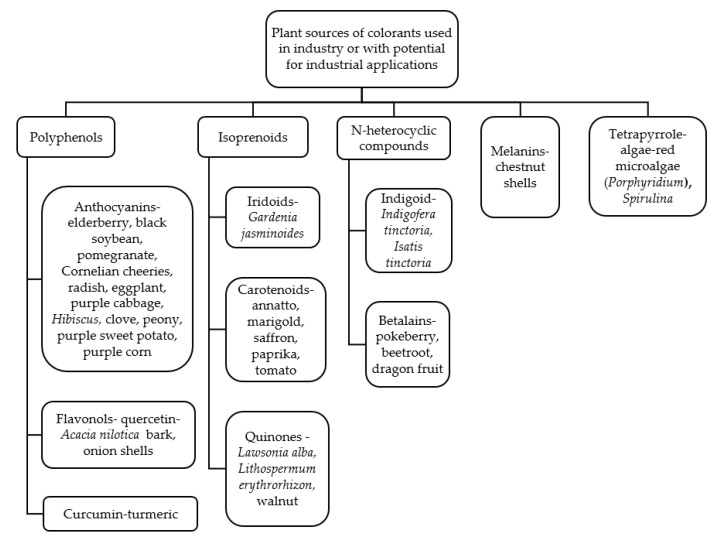
Plant sources of colorants used in the food, textile or cosmetic industries or with potential for food, textile or cosmetic industry applications.

Although most biomolecules are colorless, there is a wide range of colors available in nature that can be used in various areas of industry where there is interest in stable plant-derived colorants. In line with today’s increasing societal desire to return to natural ingredients, plant-derived dyes could be used to some extent and in certain types of industry for example in cosmetics, food and textile an alternative to synthetic colorants. Figure 2 shows various plants containing different groups of compounds that can be a source of colorants either already used in or with potential for use in food, textile or cosmetic industry applications indicated in various studies referenced in this article.

## 2. Polyphenol Applications

### 2.1. Anthocyanins

The subgroup of flavonoids that is mainly responsible for giving color comprises anthocyanins [4]. Plants that are rich in anthocyanins include blackcurrant, blueberry, raspberry, wild rose, elderberry, red grapes, chokeberry, blackberry, cornflower and black corn. Anthocyanins are also found in the petals of hibiscus flowers or mallow and the leaves of red cabbage, onion or rhubarb shoots. They are present in fruits, flowers, leaves and even storage organs. In a number of plants, concentrations of these compounds range from 0.1% to 1% dry weight [4]. Anthocyanins are extracted from plants by liquid–liquid extraction, such as by the use of organic solvents, which are used for many years, but often are toxic or not environmentally friendly [9]. Newer methods are for example supercritical fluid CO_2_ extraction, ultrasound extraction or the application of hydrolytic enzymes [9], but alternative methods of obtaining those colorants are also being developed by scientists, one example being the fermentation of vegetable matrices [9]. In some plants like *Panax ginseng* or *Camellia japonica*, there is only one type of anthocyanin, but there is usually a mixture of various types in plants [4]. About 50% of anthocyanins in nature include cyanidin moiety and the most commonly occurring sugar molecule is D-glucose [10]. Functional groups like hydroxyl, methoxyl, acyl or glycosyl attached to the structure of anthocyanins strongly affects its stability. Moreover, monoglucosides are less stable than diglucosides, but diglucosides containing additional sugar molecules undergo browning reactions [4]. Additionally, the amount of hydroxyl and methoxyl groups present in anthocyanin molecules affect color: The hydroxyl group gives more of a bluish shade, while methoxyl imparts more of a reddish hue. These functional groups are not the only to affect the color of anthocyanins, as medium conditions or interactions of anthocyanin molecules with other molecules may also influence the shade. The main issue with anthocyanins and other botanical colorants is their instability, which in practice means losing their color. Anthocyanins are sensitive to light, oxygen and temperature, and thus they have to be stored in low temperatures, without light and with low oxygen availability [4]. 

To enhance color stability of anthocyanins, short-term and long-term factors may be applied. The former concerns copigmentation and self-association, while the latter relates to creating polymeric colorants or pyranoanthocyanins [11]. Pyranoanthocyanins are found in red wine, which is characterized by very good stability of color over time. For this reason, its chemical structure continues to be examined [12]. Pyranoanthocyanins were discovered in red wine for the first time in the year 1996 as ingredients responsible for its long-lasting color [13,14]. They are anthocyanin derivatives containing an extra pyran ring, which stabilizes the structure and imparts a more orange color compared to anthocyanins colorants [14]. The natural formation of those stable compounds is a long process requiring time and is therefore inefficient and not practical in industry applications. In consequence, studies aiming to accelerate the formation of pyranoanthocyanins through a reaction of heated anthocyanins and cofactors have been conducted and reported by different authors. Straathof and Giusti [12] indicated that pyranoanthocyanins can be obtained from heated elderberry anthocyanins and caffeic acid efficiently over time and may have the potential to be applied as stable food colorants. Copigmentation is observed in plants, in which organic compounds not responsible for their color and pigments create complexes or intermolecular bonds giving plants more intense colors than the concentration of pigments would suggest [3]. This copigmentation phenomenon is also called intermolecular copigmentation. The next, more effective type is intramolecular copigmentation, which is correlated with the acylation of anthocyanins. Acylated anthocyanins do not undergo rapid hydration reactions. These reactions depend on pH values and lead to loss of color. They show better resistance to heat, light and other factors. In both types of copigmentation, copigments protect the color of anthocyanins by impeding the nucleophilic attack of the water molecule [4]. Many substances have been recognized as copigments, such as chlorogenic acid, sinapic acid and ferulic acid [3]. Furthermore, adding copigments attracts more interest in the food industry to increase the color stability and diversity of products.

Anthocyanins usually are used as red colorants in food, but their degradation restricts their application in industry. However, several studies have indicated the possibility of using anthocyanins as food colorants instead of artificial dyes. In one study, a copigment like sinapic acid was added to anthocyanin obtained from black soybean and then nanoencapsulated with the usage of the ionic gelation method to examine the influence these processes have on color stability and the antioxidant activity of colorant [15]. Both copigmentation and encapsulation processes enhanced the color and antioxidant activity of anthocyanin. Ko et al. suggested that a combination of these two methods could be used in practice, such as in the food industry with the double benefit of not only a stable product color but also antioxidant activity [15]. Pangestu et al. demonstrated that non-acylated anthocyanins such those obtained from *Sambucus peruviana* and copigmented with ferulic acid could be potentially applied in commercial food products [16]. An improvement of the copigmentation effect was also achieved with pomegranate extract [17]. Anthocyanins as colorants are often used at a low pH, below 4, but some anthocyanin 3-glucosides, such as malvidin 3-glucoside, are relatively stable at a higher pH and can be used for alkaline products [4]. A number of studies have been conducted to obtain long-lasting, stable colors of anthocyanins in less acidic conditions [10] due to their instability in neutral and alkaline solutions. Food applications can also be found for acylated vitisins from *Vitis vinifera* or malonylated anthocyanins found in purple sunflower seeds, which contain red cyanidin derivatives [4]. Anthocyanins present in black carrot are one of the most color stable among all anthocyanins applied in the food industry due to the presence of acylated groups. Another source of anthocyanins for food applications includes Cornelian cherries acting as red colorants. According to Moldovan and David [18], they are stable during storage and show color resistance to organic food preservatives like potassium sorbate and sodium benzoate. Due to their satisfying stability, tinctorial power and coloring properties, anthocyanins that consist of pelargonidin [19,20] present in radish could be an alternative to synthetic colorants like allura red [21], which is nearly the most frequently consumed dye. Byproducts of red radish fermentation were also investigated by Jing et al. as a potential source of natural colorants [22]. Another anthocyanins-rich source with potential for food industry applications is eggplant [23]. 

Apart from (i) the ability of anthocyanins to impart color to food products or (ii) the antioxidant properties that anthocynanins have, they can also be applied in food packaging [9]. Anthocyanins, which are pH sensitive, can act like biosensors when incorporated into biodegradable polymer films [9]. This could help to monitor the freshness of various raw foods like fish or meat, which was examined in the studies. In these studies, extracts from the *Hibiscus* flower were used [24,25]. Other research conducted by Pereira et al. [26] indicated that purple cabbage *Brassica oleracea* extracts containing anthocyanins, when added to chitosan films, can be useful in creating intelligent food packages acting as a temperature indicator. 

In addition, anthocyanins can be used in fabric dyeing. For instance, their dyeing and antibacterial properties were examined in textiles (silk, wool and cotton). Fabrics dyed with anthocyanin water extracts obtained from clove, pomegranate and peony showed very good antimicrobial activity against *Staphylococcus aureus* [27]. 

It is interesting to note that anthocyanins may also be adsorbed on inorganic compounds. For example, the adsorption of anthocyanins on metal oxide like TiO_2_ is highest at pH = 3–4 [28]. Moreover, the copigmentation of anthocyanins with caffeine increases the number of red flavylium cations and blue quinoidal bases at middle pH values [10,29,30]. This effect enhances the concentration of the colored form of anthocyanins at the pH level where dye adsorption on TiO_2_ is highest [31]. This observation can be used to produce insoluble compounds consisting of plant-derived dyes and inorganic substrates for cosmetic application. Furthermore, plant extracts rich in anthocyanins have been applied as colorants in lipstick formulation [32]. The cosmetic color obtained was acceptable, and the most stable were formulations mainly containing cyanidin derived from purple sweet potato, purple corn and elderberry. An accelerated test of colorant properties indicated over 2 years of shelf stability and showed that anthocyanins could be applied as colorants in lipid-based cosmetic products [32]. 

Scientific interest in anthocyanins is significant. According to the Scopus^®^ Database, over the past 10 years, about 5949 papers have been published in which the word «anthocyanin» appears in the title of an article. When the search is extended to include the article title, abstract and keywords, results show that 22,749 papers have been published. Additionally, when the search is extended with the word «colorant», «dye» or «pigment», there are «513», «780» or «3776» papers published, respectively (these data were collected in May 2021). Most likely, there are also reports not provided by Scopus^®^, and thus they cannot be accessed easily. The research on anthocyanins is neither a closed nor a completed topic and the number of scientific reports will be increasing. However, data collected from the Scopus^®^ Data Base show that anthocyanins are not commonly investigated for use as colorants.

### 2.2. Flavonols-Quercetin

One member of the flavonol compound group is quercetin, which can be used to dye textiles various colors, particularly cotton. Quercetin, present in extracts of *Acacia nilotica* bark (kikar), has been used to dye UV-irradiated cotton fabrics [33]. In this research, textiles and plant extract were exposed to UV radiations for 30 to 120 min. Colorant was extracted both from irradiated and un-irradiated plant powder and then used to dye irradiated and un-irradiated cotton. Various parameters of the process were applied like different dyeing times, liquor concentration, electrolyte concentration, pH and temperature. Mordants like tannic acid, copper sulphate, iron sulphate and aluminum sulphate were added. Colorfastness under the influence of rubbing, washing and light was determined to assess the impact of UV radiation. The results provided by Adeel et al. [33] indicated that 50 min of dyeing at a temperature of 55 °C at pH of 6 with iron salt mordant in a concentration of 8 g/100 mL was the most optimal and gave satisfying colorfastness. Other studies have shown that exposing cotton to UV radiation for 90 min modified its surface [33]. Another source of quercetin—the powder of onion shells—was also used to dye cotton fabrics. As in the previous research study, the dye was extracted from both an irradiated and un-irradiated onion shell powder, which was then used to dye irradiated cotton fabrics. To improve the colorfastness and strength of the textile, iron and alum were added as pre- and post-mordants. Satisfying colorfastness and maximum strength were achieved at a temperature of 60 °C with a liquor ratio of 1:30 and with alum as a pre-mordant in a concentration of 10% and as a post-mordant in a concentration of 6%. Research conducted by Rehman et al. [34] indicated that gamma irradiation enhanced the colorfastness and color strength of quercetin-dyed cotton. 

Scientific interest in flavonols is also considerable. According to the Scopus^®^ Database, within the past 10 years, approximately 1080 papers have been published in which the word «flavonol» appears in the title of an article. When the search is extended to article title, abstract and keywords, results shows that 8411 papers have been published. Additionally, when the search is extended with the word «colorant», «dye» or «pigment» there are «23», «75» or «385» papers published, respectively (these data were collected in May 2021). Data collected from the Scopus^®^ Database show that flavonols are not commonly studied as a source of color.

### 2.3. Curcumin-Turmeric

The turmeric rhizome is appreciated as both a spice plant and a colorant used in food and cosmetic production. Curcumin, demethoxycurcumin and bisdemethoxycurcumin are responsible for its yellow color. Curcumin is an odorless, yellow, crystalline powder slightly soluble in water and soluble in ethanol and methanol. Ground turmeric is stable at moderate temperatures; in this form, it can be stored for almost 6 months, though it is susceptible to microbiological hazards [4]. Water activity does not affect its stability to a significant degree, but it degrades in an alkaline environment and is light-sensitive. This negative effect can be reduced by the addition of aluminum ions. In one study, the stability of curcumin added into food product (extruded and expanded balls made from soybean and corn flours) was examined and compared to tartrazine. At ambient conditions, product shelf life was measured to be 6 weeks. Besides the fact that turmeric colorant degraded faster than tartrazine, the authors of the study mentioned suggested that curcumin could be an alternative to synthetic colorant used in extruded food [35]. Turmeric can also be used as a colorant or biomordant in textile dyeing. Turmeric powder was applied on cotton fabrics, which were then dyed with black carrot leaf extract. Results provided by Batool et al. [36] indicated that turmeric biomordant led to higher color strength of the textiles tested. The effect was achieved by the formation of an additional hydrogen bond between hydroxyl groups of cellulosic fabric and anthocyanin bonded with biomordant. In the case of cotton, a more intense color was obtained during the application of turmeric biomordant than during the application of a metal dye complex. A 2% concentration was optimal in the pre-mordanting process, while in the post-mordanting process, 8% yielded optimal results. Higher concentrations of turmeric biomordant could contribute to precipitation while lower concentrations may not lead to sufficient dye binding with the textile. Treating the dyed textile with washing, light and heat yielded acceptable results [36]. Other studies concerning the use of turmeric in textile dyeing were conducted by Sachan and Kapoor [37]. Different methods of extracting yellow colorant from turmeric led to the conclusion that spray drying yields the purest dye while solvent extraction produces maximum yield. Two percent of the colorant used was optimal to efficiently dye silk, wool and cotton with various mordants giving different shades of the color [37]. 

Scientific interest in turmeric is smaller than in the case of anthocyanins. According to the Scopus^®^ Database, within the past 10 years, roughly 1512 papers have been published in which the word «turmeric» appears in the title of an article. When the search is extended to article title, abstract and keywords, results show that 5139 papers have been published. Additionally, when the search is extended with the word «colorant», «dye» or «pigment» there are «56», «279» or «241» papers published, respectively. Within the past 10 years about 12,106 papers have been published in which the word «curcumin» appears in the title of an article. When the search is extended to article title, abstract and keywords there are 23,957 papers published. Additionally, when the search is extended with the word «colorant», «dye» or «pigment» there are «63», «558» or «417» papers published, respectively (these data were collected in May 2021). Nevertheless, curcumin-turmeric-based colorant has some potential to be applied in several fields. 

## 3. Isoprenoid Applications

Isoprenoids are also called terpenoids and are divided into three groups: Iridoids, carotenoids and quinones. According to the Scopus^®^ Database, within the past 10 years about 621 papers have been published in which the word «isoprenoid» appears in the title of an article. When the search is extended to article title, abstract and keywords there are 5341 papers published. Additionally, when the search is extended with the word «colorant», «dye» or «pigment», results show that «10», «33» or «297» papers have been published, respectively (these data were collected in May 2021). However, within the group of terpenoids there are iridoids, carotenoids and quinones, which should be characterized separately. 

### 3.1. Iridoids

The most popular plants containing iridoids are dicotyledonous plants like *Gardenia jasminoides*, whose ripe fruits are widely used as a natural colorant. This plant can be a source of blue, yellow and red pigments, which have been successfully used in textile, cosmetic and food industries for many years [38]. The most significant and principal iridoid glucoside obtained from *Gardenia jasminoides* is geniposide. The reaction of geniposidic acid with amino acids like glutamic acid and arginine with the addition of citric acid yields a purple-red polymer, which is soluble in water, stable in a wide range of pH, but light-sensitive. More light-resistant are polymers containing geniposide and glycine [4]. Geniposide aglycon part- genipin can be obtained in reaction with β-glycosidases. Genipin reacts with amino acids forming blue pigment [38]. Studies related to optimizing the production parameter of the one-step chemoenzymatic conversion of geniposide into blue pigment by different glycosidases were conducted by Cho et al. [38]. Among various tested amino acids, tyrosine and glycine made it possible to obtain colorants with the highest production yields. The molar ratio of genipin and amino acids that was the most optimal was unity. Thus, blue pigments obtained in this study were used to dye fabrics like wool, silk and cotton. The examined fastness of colorants was quite good even though no mordants were applied. Adding mordants could improve the color properties of dyed textiles. Compared to other plant-derived dyes, color yields of the colorants obtained were described as much higher [38]. 

Within the last 10 years, iridoids have mainly been studied as colorants in the textile and food industries. The total number of papers in which the word «iridoid» appears in the title of an article according to the Scopus^®^ Data Base is 586. When the search is extended to article title, abstract and keywords, results show that 3762 papers have been published. Additionally, when the search is extended with the word «colorant», «dye» or «pigment» there are «13», «43» or «34» papers published, respectively (these data were collected in May 2021). So, there is a small number of studies concerning iridoid used as colorants.

### 3.2. Carotenoids

Carotenoids are terpenoids created by eight isoprenoids units and produced by the isoprenoid pathway. Carotenoid names are commonly connected with the plant from which they were isolated for the first time. As an example, β-carotene was isolated for the first time from carrot in the year 1831. Carotenoids, responsible for giving colors like pink, red, yellow, and orange, are commonly found in nature in almost every organism. In plants, they occur in plastids in leaves, fruits, flowers, but not commonly in roots [4]. Carotenoids occur in plants in the amount of approximately 0.07–0.2% dry mass. Only in leaves do carotenoids occur in free form; in other tissues, they are esterified. They also occur in combination with proteins creating noncovalent complexes. Carotenoids, called isoprene lipids, are soluble in nonpolar solvents [4]. Sources of carotenoids include tomato, annatto, carrot, peach, pumpkin, red palm oil, paprika, saffron and wild rose. For centuries, carotenoids were used as natural dyes. Besides their dying properties, they are also antioxidants. In textiles, food and cosmetics—in self-tanners for example—various carotenoid sources can be applied. 

Within the last 10 years, according to the Scopus^®^ Database, about 5703 papers have been published in which the word «carotenoid» appears in the title of an article. When the search is extended to article title, abstract and keywords, results show that 29,840 papers have been published. Additionally, when the search is extended with the word «colorant», «dye» or «pigment» there are «325», «343» or «9865» papers published, respectively (these data were collected in May 2021). Despite such a large number of studies on carotenoids, studies focused on their coloring properties are in the minority.

#### 3.2.1. Annatto

One of the most widely used carotenoid sources is annatto, which contains norbixin and bixin and can be used as a water-soluble extract, an oil soluble extract or oil suspension. Annatto colorant imparts a red or orange hue and often has good stability, but its color depends on pH. Annatto has long been used as a colorant for food, such as in dairy products, and has had applications in cosmetics, pharmaceuticals and textiles, where examples of the last category include silk, wool and cotton. Studies concerning dyeing polyester and nylon fibers with annatto were conducted by Gulrajani et al. [39]. Research showed that these synthetic fibers were compatible with annatto colorant. At higher temperatures, dye absorption increased as the process was endothermic. The color was resistant to washing but was light sensitive. Moreover, research showed that different mechanisms were used to dye ionic and non-ionic textiles [39]. In other studies, the extract of annatto seeds was used to dye cotton and silk fibers, and both materials showed good fastness properties. Meena Devi et al. [40] also suggest that annatto dye can be desirable for dyeing Band-Aid cloth due to its antimicrobial properties, which may have a positive influence on wound healing. Likewise, annatto has been used as a source of colorant in cosmetic products. Different amounts of annatto extract were added to lipstick formulas in studies conducted by Aher and Bairagi [41]. As a result, color varied from yellow-red to light red to dark red. Light red lipstick was rated the best among test specimens and met with a 60% approval of the testers [41]. 

Within the last 10 years, according to the Scopus^®^ Database, about 192 papers have been published in which the word «annatto» appears in the title of an article. When the search is extended to article title, abstract and keywords, results show that 400 papers have been published. Additionally, when the search is extended with the word «colorant», «dye» or «pigment» there are «63», «112» and «72» papers published, respectively (these data were collected in May 2021). 

#### 3.2.2. Marigold

Another carotenoid source is marigold, an annual herb containing lutein whose dried flower petals are used for dyeing. Marigold is a source of a heat stable and light stable dye of high tinctorial power, which can be mixed with vegetable oil, gelatin or calcium silicate. Additionally, price prevails in favor of marigold in comparison to other yellow and orange dyes like saffron and turmeric. Proper storage of marigold flowers is crucial for dye stability. This stability can be affected by promising techniques such as anaerobic and lactic acid treatment. Marigold petals are used as a food additive and as a textile colorant [4]. Extracts of marigold are also applied in poultry feed to enhance the color of egg yolks. In a study by Sowbhagya et al. [42], dried marigold petals were added to butter creams, where this procedure yielded promising results as the shelf life and quality of the food products tested improved [42]. The dye obtained from marigold was also used by Rashdi et al. [43] to dye lyocell fabric with various metallic salts with two mordanting methods. The best dyeing results were obtained for ferrous sulfate acting as a mordant with the post-mordanting method, and the study showed that marigold colorant did not affect the structure of fibers. Another study concerned dyeing cotton fabrics with marigold supported by the ultrasound method. The textile was dyed yellow and, compared to conventional dyeing water, salt concentrations and thermal energy were saved [44]. 

Within the last 10 years, according to the Scopus^®^ Database, about 543 papers have been published in which the word «marigold» appears in the title of an article. When the search is extended to article title, abstract and keywords, results show that 1335 papers have been published. Additionally, when the search is extended with the word «colorant», «dye» or «pigment» there are «19», «58» and «93» papers published, respectively (these data were collected in May 2021). 

#### 3.2.3. Saffron

Saffron is obtained from dried stigmas of *Crocus sativus* cultivated in countries such as Iran, India and Spain and has been used since ancient times [45]. Its main active compounds are safranal, crocin and picrocrocin. Safranal is responsible for the aroma of saffron, while picrocrocin gives it a bitter taste. Crocin is a carotenoid that gives saffron its yellow-orange color. It is easily water soluble, which is a great advantage because other carotenoids require a special formulation to be soluble or dispersible in aqueous media [4]. Saffron is characterized by high coloring power and good stability; additionally, it does not fade quickly. Solutions of saffron are largely stable in acidic and alkaline mediums thanks to the content of dicarboxylic acids, esters, nitrogen compounds and crocin [45]. For a long time, saffron has been used in food, medicine, painting and textiles. Its buffer solutions reduce cellulose oxidation [46]. Saffron has also been applied as a colorant in cosmetic products, one example being its use to dye lipstick. According to the current trend focused on incorporating natural raw materials into cosmetics, there is a chance for saffron to be used once again in cosmetics, but rather in small amounts due to its high cost [45]. Saffron can be used the same as turmeric or can be a botanical alternative for tartrazine. Synthetic fabrics were dyed by Elmaaty et al. [47] with saffron and curcumin. Prior treatment with UV/Ozone for varying durations of time brought about an improvement in the dyeing process for both colorants. 

Within the last 10 years, according to the Scopus^®^ Database, about 1310 papers have been published in which the word «saffron» appears in the title of an article. When the search is extended to article title, abstract and keywords, results indicate that 2238 papers have been published. Additionally, when the search is extended with the word «colorant», «dye» or «pigment» there are «44», «93» and «63» papers published, respectively (these data were collected in May 2021).

#### 3.2.4. Paprika

Another source of carotenoids is the deep red powder obtained from the dried pods of sweet paprika, which contain capsanthin and capsorubin. Paprika oleoresins are used in the food industry. The properties of encapsulated paprika oleoresin in rice starch/gelatin and Arabic gum were evaluated by Santos et al. in food applications, with a satisfying effect without affecting its texture, taste or flavor [48]. 

Within the last 10 years, according to the Scopus^®^ Database, about 309 papers have been published in which the word «paprika» appears in the title of an article. When the search is extended to article title, abstract and keywords, results show that 674 papers have been published. Additionally, when the search is extended with the word «colorant», «dye» or «pigment» there are «23», «51» and «81» papers published, respectively (these data were collected in May 2021). 

#### 3.2.5. Tomato

Another carotenoid source is tomato, which contains lycopene and β-carotene. Research conducted by Yildiz and Baysal [49] showed that to increase lycopene content and thus enhance the color of tomato puree, electroplasmolysis can be used during the preheating process. This process made it possible to achieve a better color of tomato puree in comparison to steam methods [49]. 

Within the last 10 years, according to the Scopus^®^ Database, about 19,808 papers have been published in which the word «tomato» appears in the title of an article. When the search is extended to article title, abstract and keywords, results show that 37,110 papers have been published. Additionally, when the search is extended with the word «colorant», «dye» or «pigment» there are «34», «227» and «922» papers published, respectively (these data were collected in May 2021). 

#### 3.2.6. Improving the Properties of Carotenoid Colorants

Carotenoids are sensitive to light, temperature, pH and redox agents. An effective method to improve their properties is to microencapsulate carotenoids like β-carotene, which extends its shelf life and modifies the color [46]. In one study, β-carotene was uniformly dispersed in a shellac polymer matrix to prevent its degradation over time. Different concentrations of β-carotene were loaded to observe color change. Moreover, adding antioxidants like caffeine or propyl gallate improved β-carotene stability. Shellac particles have the ability to implement various hydrophobic colorants, therefore this process can be useful in natural coloring in industrial applications [46]. It has the potential to be applied in cosmetic products as well. As few naturally occurring carotenoids are water soluble, with crocin as the main representative, many attempts have been made to obtain a larger number of hydrophilic carotenoids. Converting carotenoids into water-soluble molecules entails adding very polar groups; for instance, the presence of a carboxyl group in crocetin and bixin contributes to it being slightly water soluble [50]. Carotenoid hydrophobicity is a disadvantage in industrial applications, where they can be used as pharmaceutical antioxidants or food colorants. Various methods have been developed—usually patented—to improve the water solubility of carotenoids. Mainly physical methods such as dispersion in polyethylene glycol were used, but chemical derivatization was also used, two examples being complexation with cyclodextrins or carotenoid glycoside synthesis [50]. To obtain water-soluble powders carrying agents such as acacia gum or modified starch may be required [4]. The process of enhancing the hydrophilic properties of some carotenoids can be beneficial for other reasons as well. For example, there is an increase in the antioxidant activity of water-soluble carotenoids compared to the same hydrophobic carotenoids in an organic solvent [51]. Carotenoids are also examined for their stability. Carrot concentrates are used to produce powders containing crystalline carotenoids. They were examined by Haas et al. [52] for stability during drying and storage. For this purpose, various stabilizing methods were checked, such as oxygen-free storage, freeze and spray drying or different additives (gum Arabic, maltodextrin, modified starch, sodium ascorbate and tocopherols). All methods showed low carotenoid loss, with less than a 5% loss of carotenoids during the processing stage [52]. These conclusions could facilitate industrial applications of carotenoids and make them more practicable for mass production.

### 3.3. Quinones

The quinone spectrum of color is very wide, ranging from deep purple to orange and yellow. Quinones have been important colorants since ancient times. The most well-known quinones include alizarin, rubiadin, xantopurpurin, purpurin, alkanna, chrysarobin and cochineal. Naphthoquinone-lawsone from *Lawsonia alba* is widely used in cosmetic formulas for hair dyeing [4]. Another example of naphthoquinone is shikonin, which was the first product obtained from cell cultures of *Lithospermum erythrorhizon* and was used in 1985 by the Japanese cosmetic company Kanebo to produce lipstick, this arousing great interest in the cosmetic market [53]. The next representative of naphthoquinones is juglon. Found in walnuts, juglon can be applied in cosmetic formulas such as hair dyes. Walnut extract with the addition of iron sulfate, aloe extract and ascorbic acid was used by Beiki et al. [54] to color hair. In this study, dark brown dyed hair was resistant to light and washing. Color strength was acceptable, and the hair dye obtained did not cause irritation [54].

Anthraquinones like alizarin and purpurin are present in *Rubia tinctorum* roots, which are used to dye textiles such as cotton and linen. Anthraquinones from insects (kermes and cochineal) were used as textile dyes and food colorants. Cochineal has long been known, as it was used by the Aztecs. Cochineal is obtained from dried insects, whose carmine content is approximately 22%. From 80,000 to 100,000 insects are needed to obtain 1 kg of dye [4]. Previously, colorant extraction was performed with hot water, while currently ethanol is used. In the next step, the solvent is removed, and the concentrated solution is approximately 2–4% carmine acid. The color of cochineal does not directly depend on the amount of carmine acid, but rather is an indicator of colorant quality [4]. Carmine acid is water soluble and has the ability to react with different types of metals, yielding pigments that are insoluble in water. These pigments are called carmines and are stable under the influence of light; they are, however, susceptible to changes in temperature, pH value and UV radiation. Very often carmines are mixed with maltodextrin, polypropylene glycol, glycerin, citric acid and sodium citrate. Lac and kermes have structures similar to carmine acid. Kermes is obtained from insects living on some species of oaks [4]. Cochineal is a widely used stable colorant but, despite all the advantages listed, it can cause allergies. For this reason, attempts have been made to replace it in cosmetic products with plant-derived colorants. The cosmetic brand Chanel collaborated with the company CRITT Horticole of Rochefort to obtain a new cosmetic alternative and create an insoluble coloring substance. Plant extracts containing different coloring substances are mainly soluble dyes, but to obtain insoluble pigments, which are required for make-up applications, Chanel and CRITT fixed colored plant extracts onto an inorganic substrate. From the list of plant species imparting red coloration, sappan wood was selected with mineral support from kaolin. The choice was dictated by different parameters such as color strength, spectrocolorimetric measurements, dispersibility in a powder medium, toxicological data, uniformity during the application and wettability in a lipophilic medium. Analysis was also performed on stability of obtained product under the influence of light and temperature, as well as on its anti-oxidative and anti-aging properties. As a result of this research, a red pigment was obtained for lipstick, glosses, eyeshadows and blushes, one that is based on the sappan wood extract on a kaolin substrate [55]. It is worth mentioning that plants and animals are not the only source of anthraquinones. They can also be found in marine-derived fungi [56]. Usually, anthraquinones in fungi are biosynthesized by one of two known pathways proceeding with the formation of intermediate product-polyketide. This group includes azaphilone colorants obtained from the *Monascus* fungi, which are widely applied in different branches of the dyeing industry, as well as in the food, textile and cosmetic industries, due to their UV radiation absorbing properties [56,57].

Scientific interest in quinones is impressive. Within the past 10 years, according to the Scopus^®^ Database, about 3088 papers have been published in which the word «quinone» appears in the title of an article. When the search is extended to article title, abstract and keywords, results show that 20,991 papers have been published. Additionally, when the search is extended with the word «colorant», «dye» or «pigment» there are «18», «428» or «606» papers published, respectively (these data were collected in May 2021). As with the other groups of compounds, despite such a large number of studies on quinones, studies focusing on their coloring properties are in the minority.

## 4. Applications of N-Heterocyclic Compounds 

### 4.1. Indigoid

The N-heterocyclic compound group includes one of the oldest colorants known to humans, indigoid, which is important in dying textiles, especially wool. It is obtained from *Indigofera tinctoria* and *Isatis tinctoria*. Indigo is one of colorants that does not need mordants. Presently, it is used more locally; not on a large scale, but rather in craft dyeing. 

Within the last 10 years, according to the Scopus^®^ Database, about 31 papers have been published in which the word « indigoid » appears in the title of an article. When the search is extended to article title, abstract and keywords, results show that 140 papers have been published. Additionally, when the search is extended with the word «colorant», «dye» or «pigment» there are «10», «102» or «25» papers published, respectively (these data were collected in May 2021). Despite a small number of studies indigoids, most of them focus on indigoid’s coloring properties.

### 4.2. Betalains 

Betalains are derivates of betalamic acid and among them, two groups can be distinguished: Purple-red betacyanins and yellow betaxanthins. More than 50 betalains occurring in plants have been described. These types of colorants are present in 13 families of the order *Caryophyllales* in fruits, flowers, stems, roots, bracts and in some genera of higher fungi [5]. Various betalain sources are edible, such as red beetroot, dragon fruit, cacti fruit, amaranth and radish [58]. These colorants have a high tinctoreal capacity and high molar absorptivity, thus a small amount of pure betalains is enough to obtain the desired hue [4]. Betalains show antioxidant activity, which is very well documented by diverse methodologies in several studies. Betacyanins display better antiradical properties than betaxanthins. It has also been observed that their presence is connected with the occurrence of proteins with antifungal properties [4]. Betalains are considered to be generally more stable than anthocyanins [59]. In contrast to anthocyanins, betalains are stable in a broader pH range and possess the ability to regenerate after thermal treatment [5]. However, their stability can be affected by multiple factors, including a pH lower than 3 and higher than 7, high water activity, metal cations, light and high temperatures [60]. The stability of betalains is higher in beetroot juice than in pure extracts. It also increases when water activity is low, as water is the main factor in color loss; for this reason, the powder form of betalains is more stable over time than the water extract [4]. Acylated betalains demonstrate better stability than nonacylated ones because they do not undergo racemization. 

Since the beginning of the 20th century, betalains have been used as food colorants. Pokeberry juice, for example, was added to red wine to improve its tint [4]. In industry, betalains are applied as a juice or as a powder that is obtained mainly by extracting water from beetroots. Such products have a betalain content of 0.3–1%. Betalains from beetroots are used as pink or violet pigments in food products such as jams, fruit yogurts, ice-creams and many others. Additionally, the same coloring substance may also be used in pharmaceuticals and cosmetics [58]. Efforts to obtain color cosmetics with betalains as coloring agents were made by Azwanida et al. [61]. Namely, a lipstick was created with betalain pigments extracted from dragon fruits as an alternative to synthetic colorants, thereby minimizing side effects like allergies or dry lips. Four percent dragon fruit extract was added to the lipstick formula. At room temperature, the lipstick color was considered stable, but after one month of storage at a temperature of 40 °C, the color underwent a drastic change from red to darker red. Additionally, the antioxidant properties of formula were measured. Antioxidant potential after the addition of betalains pigments increased, which might be also attributed to a significant amount of phenolics compounds in the dragon fruit extract. Lipstick formulated with betalain pigments extracted from dragon fruits displayed satisfying levels of coverage, spreadability, smoothness and stability when stored at room temperature [61]. Thus, using plant-derived colorants may undoubtedly increase customer acceptance. Amaranth contains compounds belonging to the betacyanins group, among them amaranthine dye. Due to acylation, their stability is higher than those extracted from radish. It is a water-soluble colorant found in different parts of the plant (obtained from fresh leaves) that could be applied to dye food such as drinks. The amaranth juice has also been used by women as a facial cosmetic (a rouge) [62]. Numerous studies have been conducted to increase betalains’ potential for industrial applications. To improve stabilization, ascorbic acid, isoascorbic acid or gluconic acid are added. Studies focusing on the encapsulation of natural colorants such as betalains to enhance their color stability may yield new possibilities that are worth considering [5]. Betalains, for instance, can be blended with various chemical substances. One example concerns blending betanin and betanidin with α-tocopherol, then incorporating them into liposomes to observe additional effects and improved properties [63]. 

Within the last 10 years, according to the Scopus^®^ Database, about 143 papers have been published in which the word «betalain» appears in the title of an article. When the search is extended to article title, abstract and keywords, results show that 634 papers have been published. Additionally, when the search is extended with the word «colorant», «dye» or «pigment» there are «95», «82» or «362» papers published, respectively (these data were collected in May 2021). Despite a small number of studies on betalains, most of them focus on their coloring properties. 

## 5. Melanin Applications

Another type of naturally occurring colorant is the melanin group. They are polymers built by phenolic or indolic monomers that can be complexed with proteins or carbohydrates, imparting brown, grey and black hue to animals and plants. Brown chestnut shells have an herbal melanin content of approximately 15%. They are food industry byproducts and can be used as antioxidative food colorants [64,65,66]. Although it is not a dye of plant origin, it is worth adding that the ink of cuttlefish *Sepia officinalis* is blackened by melanins and has long been used by humans for different purposes, one of them being to dye food [67]. Black ink from *Sepia officinalis* can also be used as a colorant in cosmetics, this being examined by Neifar et al. [68] in mascara and eyeshadows. Satisfactory results were obtained: The addition of these black ink to iron oxide and black bone dyes increased spreadability, covering capacity and the color of make-up cosmetics [68]. 

Within the last 10 years, according to the Scopus^®^ Database, about 2196 papers have been published in which the word «melanin» appears in the title of an article. When the search is extended to article title, abstract and keywords, results show that 13,324 papers have been published. Additionally, when the search is extended with the word «colorant», «dye» or «pigment» there are «29», «226» and «2915» papers published, respectively (these data were collected in May 2021). According to the data collected, coloring properties of melanins are not the main focus of research.

## 6. Tetrapyrrole Applications

Tetrapyrrole structure can be both linear (the basic structure is bilin) or cyclic (the basic structure is porphyrin ring). Plant bilins represented by phytochrome, phycoerythrin and phycocyanin are one of the most ubiquitous pigments on Earth [4]. Obtained from photosynthetic organism, phycobilins and phycobiliproteins are used as food colorants. For instance, phycobilins from *Spirulina* were accepted as safe colorants for chewing gum, candies, milk products and other foods. Together with chlorophyllous, they are practically the only plant-derived alternatives to blue and green color. Phycobiliproteins are used by various companies, and many patents can be found concerning both their application in the food and cosmetic industries as well as their production. However, patents relating to their application as fluorescence dyes dominate. Phycobiliproteins are stable colorants, but the protein type, the concentration thereof, purification methods, temperature and pH affect phycobiliprotein stability. In contrast to anthocyanins, different pH values do not cause a change in color of phycobiliproteins colorants [69]. One example is B-phycoerythrin extracted from red microalgae (formed by phycoerythrobilin chromophore and protein) [70]. Studies conducted by García et al. [71] indicated that B-phycoerythrin has the potential to be used as a food coloring substance in milk-based products as an alternative to synthetic dyes. Phycoerythrin could be an alternative to anthocyanins in food dyeing in pH values in which anthocyanins are no longer red. This water-soluble pink colorant may also be an interesting ingredient applied in cosmetics, textiles and pharmaceuticals [72,73,74,75]. Phycocyanin, a blue colorant, is non-toxic, non-carcinogenic and also has the potential to be an alternative to artificial dyes [76]. It is used in several countries like China, Thailand and Japan as a cosmetic colorant to dye eyeliners and lipstick or to dye food such as dairy products, candies or beverages [69]. Phycoerythrin and phycocyanin, particularly when stored dry, have a long shelf-life, which favors their use in industry. The stability of phycoerythrin and phycocyanin extracted from *Porphyridium* was studied in vitro and research showed that in temperatures around 60 °C at pH levels of 4, 5 and 6, both colorants were stable for 30 and 40 min, respectively [77]. As polyhydric alcohols and sugars have the ability to stabilize proteins, they are applied in the food industry as stabilizing agents. Research on *Spirulina platensis* has indicated that different concentrations of sorbitol can be helpful to avoid decoloration of the phycocyanin solution caused by temperature, thus confirming that color loss is associated with protein degradation [78]. In another study conducted by Jespersen et al. [79], blue food colorants like phycocyanin, indigo and gardenia blue were investigated in terms of their resistance to light and heat. Colorants were applied to various food products like soft and hard candy, jelly gum and soft drinks. Phycocyanin was the most universal colorant in the food tested, giving soft candy and jelly gum a blue color despite the fact that stability under the influence of light and heat was low [79]. 

Within the last 10 years, according to the Scopus^®^ Database, about 173 papers have been published in which the word «tetrapyrrole» appears in the title of an article. When the search is extended to article title, abstract and keywords, results show 1191 papers have been published. Additionally, when the search is extended with the word «colorant», «dye» or «pigment» there are «9», «53» and «191» papers published, respectively (these data were collected in May 2021). Research concerning tetrapyrrole’s coloring properties are not usually the focus of these studies. 

## 7. Conclusions

Color is one of the main factors that attracts consumers, especially in the context of cosmetics (particularly in color cosmetics), food and textiles. Developing colored products using botanical color agents in today’s world may enhance consumer interest to an even greater extent. Plant-derived colorants such as flavonoids, anthocyanins, betalains or carotenoids are considered more beneficial for human health and the environment and are perceived as not showing any side effects when compared to artificial ones. Botanical colorants in food, textiles and cosmetics are a demanding source of color but not impossible as demonstrated in the examples provided relating to application and studies. Though plant-derived colorants are not a common source of color in the industry, various plant colorants are found in food, and also at times in textile or cosmetics products. Colorants of plant origin could be used more widely in the cosmetic, food and textile industries as a potential alternative to some synthetic colorants. The history of almost every plant-derived colorant associated with its usage for social and aesthetic purposes provides deep knowledge about civilization’s development as well as society’s changing needs and expectations. Based on this knowledge, inspiration can be drawn to create new color cosmetics, food colorants, plant dyed textiles and new innovations and industrial solutions can be implemented based on extracting the benefits of the power of plants. Because so many materials need to be colorized, plant-derived colorants may offer a simple and safe alternative to synthetic ones in several products.
